# Teaching right from wrong: Developing a model of early immune education

**DOI:** 10.1371/journal.pbio.3003314

**Published:** 2025-08-15

**Authors:** Parsa Ghadermazi, Matthew R. Olm

**Affiliations:** Department of Integrative Physiology, University of Colorado Boulder, Boulder, Colorado, United States of America

## Abstract

Early immune education mechanisms remain poorly understood. A new PLOS Biology study develops a mathematical modeling framework to provide mechanistic insights into how the infant immune system learns to distinguish beneficial from harmful microbes.

Just as mothers play a crucial role in teaching children right from wrong, the maternal immune system serves as the first teacher for an infant’s developing immune defenses. When first confronted with the vastly diverse microbial world of the gut, an infant’s immune system relies on external guidance to learn which microorganisms to fight off and which to protect. Mothers can provide this essential instruction in part through antibodies secreted into breast milk. The immune education process has profound consequences for lifelong health, and when this early teaching is unbalanced, it can result in heightened susceptibility to autoimmune disorders and atopic diseases like asthma and allergies: conditions that are becoming more prevalent worldwide. Yet despite its importance, much remains unknown about how this maternal instruction is orchestrated in vivo. A new study by Tepekule and colleagues [1] starts to address this knowledge gap by developing a mechanistic mathematical model that captures the dynamic interplay between gut microbes and the developing immune system in breastfed infants.

Secretory Immunoglobulin A (SIgA) is the major antibody found in mucosal secretions, like gut mucosa, saliva, and breast milk [2]. It functions as an immune mediator that regulates microbial communities within the mucosal tissue, including the gastrointestinal tract. This process is assumed to occur in three modes: protective masking, where low-affinity SIgA helps beneficial bacteria by increasing their adherence to the gut epithelium and shielding them from immune surveillance; neutralizing inhibition, where high-affinity binding harms pathogenic bacteria by suppressing their growth and promoting their elimination; and selective non-interaction with immunologically neutral microorganisms. This system enables SIgA to maintain intestinal homeostasis by simultaneously fostering beneficial microbial colonization and controlling pathogenic expansion.

The mathematical framework developed by Tepekule and colleagues captures how this immune teaching unfolds over time (**Fig 1**). This model assumes that immune development happens over three distinct stages to facilitate the model parameter inference: the maternal phase, the developmental phase, and the steady phase. The maternal phase takes place during early infancy, when antibodies from breast milk provide the primary guidance in distinguishing between helpful and harmful microbes. The developmental phase occurs as infants transition from exclusive breastfeeding to mixed feeding and solid foods, where both the gut microbiome and the infant’s own immune responses gradually stabilize. Finally, the steady phase occurs when the child’s own immune system takes full control. Through this process, an infant’s own SIgA response comes to closely resemble their own mother’s antibody response patterns.

**Fig 1:**
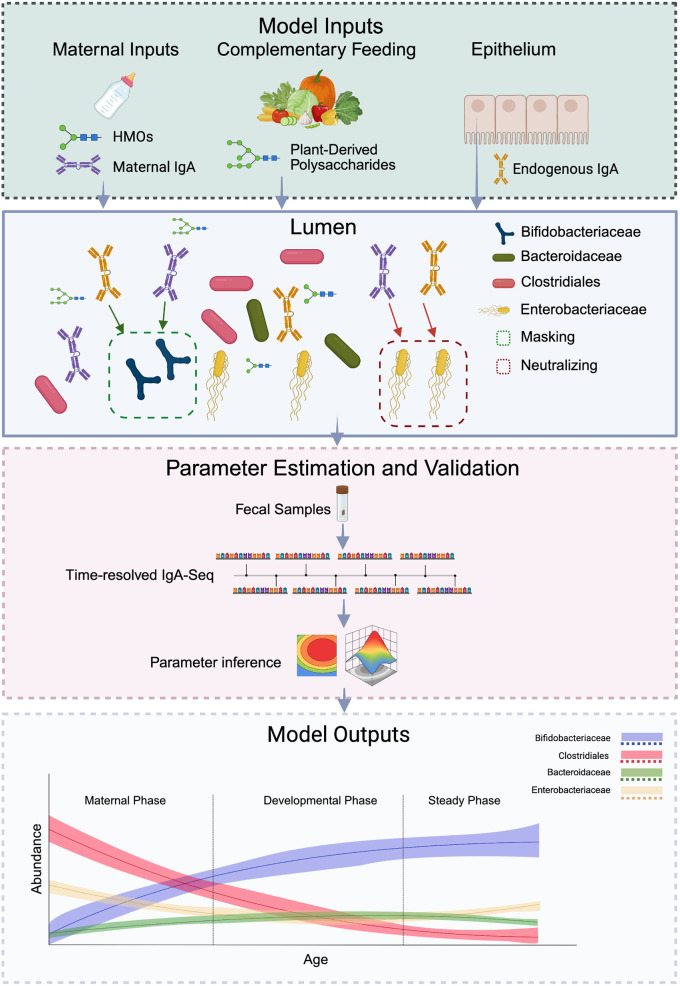
A simplified model of immune education. The model developed by Tepekule and colleagues incorporates key inputs such as maternal human milk oligosaccharides (HMOs) and IgA, infant diet, and endogenous IgA produced in the infant gut. These inputs are then modeled within the context of the infant gut lumen, where IgA can mask or neutralize microbial taxa, and HMOs and plant-derived polysaccharides selectively promote different taxa. Model parameters are informed by existing in situ longitudinal IgA-seq data [3] and infant feeding records [4]. The main outputs include the abundance and IgA binding levels of each modeled microbial taxon, allowing for predictions of how variations in inputs (e.g., changes in breastfeeding or diet) shape the developing infant microbiome.

The authors had to make several creative decisions while constructing the model to manage the exceedingly high complexity of immune education. The framework utilizes a Bayesian approach as well as grid search on datasets including sequencing data from IgA-seq experiments [3] and feeding practice records [4] for parameter calibration. They abstract all microbial taxa into just four bacterial groups that represent distinct ecological roles in infant gut development: Bifidobacteriaceae (early beneficial colonizers), Enterobacteriaceae (potential pathogens), Bacteroidaceae, and Clostridiales (families crucial for long-term digestive health). The model then runs simulations based on how antibodies interact differently with each group, sometimes acting as protective coatings that help beneficial bacteria thrive, other times functioning as neutralizing agents that suppress potentially harmful microbes. While each family contains many different species, this approach strikes a practical balance, detailed enough to capture essential immune dynamics while remaining simple enough to work with real-world data from studies tracking infant development.

The output of the model simulations reveals several crucial insights into early immune development, particularly regarding the proper balance required for proper immune education. For example, excessive abundance of the *Enterobacteriaceae* group leads to an “overactive” immune system that did not properly tolerate non-pathogenic microbes. On the other hand, simulations where the growth rate of *Enterobacteriaceae* is artificially reduced also lead to inhibited immune activation and an inability to promote beneficial microbes. The authors use this data to conclude that, during some phases of immune development, the abundance of IgA-bound *Enterobacteriaceae* can predict future immune infant health outcomes. Another of the most significant findings in the manuscript relates to how maternal immune conditions are transmitted across generations. Mothers with hyperreactive immune systems (such as those with inflammatory bowel disease) pass these tendencies to their children through the binding patterns of IgA present in breastmilk. Importantly, however, the infant’s immune overactivity was not fully inherited and was substantially reduced compared to the maternal state.

To validate the model’s findings, the researchers integrate extensive real-world data from infant fecal samples. By tracking both bacterial populations and antibody responses over time, the authors successfully test their model’s predictions against independent studies of celiac disease [5]. These validations confirm the model’s ability to identify key immune markers and distinguish healthy from disease-prone immune development patterns, potentially paving the way for new therapeutic strategies.

While this modeling framework provides valuable insights into immune education, several limitations point toward important directions for future research. The model’s focus on broad taxonomic families, while necessary given current data constraints, may obscure important species-level and strain-level differences in how bacteria interact with IgA antibodies. As more comprehensive IgA sequencing datasets become available, examining these finer taxonomic distinctions could reveal more nuanced patterns of immune-microbe interactions that are currently hidden within the broader groupings. Additionally, taxonomy itself might hide critical information about the nature of the interaction between microbes and the host [6]. Future models involving whole genome sequencing data, as opposed to the 16S rRNA amplicon data used in this model, could capture how specific bacterial genes or functions influence immune development. Further, there are several aspects of the web of interactions between the gut microbiota and the human immune system that are far too complex for any existing model to fully capture. For instance, spatial variations throughout the gastrointestinal tract are largely ignored in current modeling approaches, even though local microenvironments create distinct immune–microbe interaction patterns along the gut’s length [7]. Better techniques to measure local variability of bacterial density, immune cell populations, and metabolite concentrations need to be developed before models that account for this data can be created.

The work by Tepekule and colleagues represents a significant step forward in understanding immune education. By revealing the delicate balance required for proper immune education and demonstrating how maternal guidance shapes lifelong health outcomes, this research provides both mechanistic insights and practical implications for improving infant care and preventing autoimmune diseases. While the complexity of gut-immune interactions extends far beyond what any single model can capture, this framework establishes a foundation for future investigations that build on top of this work and address its key limitations. As we continue to unravel the intricate interaction between our immune systems and gut microbiome, such mathematical approaches prove invaluable for translating biological complexity into actionable clinical strategies.

## Abbreviations

HMOs: human milk oligosaccharides
SIgA: Secretory Immunoglobulin A
